# The complete chloroplast genome sequence of *Camellia sinensis* cv. *Dahongpao*: a most famous variety of Wuyi tea (Synonym: *Thea bohea* L.)

**DOI:** 10.1080/23802359.2020.1844093

**Published:** 2021-01-05

**Authors:** Li Li, Yunfei Hu, Linhui Wu, Rongbing Chen, Shengcai Luo

**Affiliations:** a College of Tea and Food Science, Wuyi University, Wuyishan, China; b Wuyi Turtle Rock Tea Co. Ltd, Wuyishan, China

**Keywords:** Chloroplast genome, *Camellia sinensis*, phylogenetic analysis

## Abstract

Here, combining PacBio and Illumina sequencing data, we reported the complete chloroplast genome of the first Wuyi tea (*Bohea*), *Camellia sinensis* cv. *Dahongpao* (DHP) with very high economic value. The chloroplast genome was 157,077 bp in length, with a large single copy (LSC) region of 86,633 bp, a small single-copy (SSC) region of 18,282 bp, separated by two inverted repeat (IR) regions of 26,081 bp each. It contained a total of 137 genes, with an overall GC content of 37.29%. The phylogenetic analysis showed that DHP was sister to *C. sinensis* cv. *Longjing*.

Wuyi tea has a long history, formerly known as the trade name ‘*Bohea*’ in English. Because it was the first tea exported to the West, it was also called ‘Chinese tea’ in Europe in the seventeenth century. Wuyi tea is grown in rocky soil of Mountain Wuyi in northern Fujian, China, which is a UNESCO World Heritage site and considered the birthplace of oolong tea (Xiao 2017). As the most famous variety of Wuyi tea, DHP, collected from the original bushes of its variety, is among the most expensive teas in the world, and more valuable by weight than gold (Rose 2010).

Chloroplast (cp) genome, which is highly conserved in sequence and structure due to the non-recombinant, haploid, and uniparentally inherited nature, may provide useful information to further species identification and evolutionary study (Zeng et al. 2017). For further germplasm conservation, the complete cp genome sequence of DHP was sequenced using a combination of PacBio and Illumina sequencing platforms. The Illumina data was used to evaluate the complexity of the genome and correct the PacBio long reads. Total DNA was isolated from fresh leaves of an individual of DHP, collected from Mountain Wuyi (27°43′42.46″N, 118°0′14.40″E). All the voucher specimens of DHP were preserved in the Wuyi University herbarium, and DNA samples were stored at −80 °C at the Key Laboratory of Tea germplasm Genetic Resources of Wuyi University. PacBio and Illumina reads were mapped against the published cp genomes of *Camellia sinensis* var. *sinensis* (Accession number: KJ806281) (Huang et al. 2014) using CLC Genomics Workbench 11.0.1 software to filter out the cp reads and be *de-novo* assembled by guidance-based assembly approach. To annotate the cp genome, we used the initial annotation of cpGAVAS (Liu et al. 2012) and sequenced coordinates of annotated chloroplast genes available on NCBI using BLAST search (Acland et al. 2014). Annotation errors were corrected manually. Raw reads were deposited in the NCBI Sequence Read Archive (SRA: SRX8944642 and SRX8944643) and the final annotated cp genome sequence was deposited to NCBI GenBank (Accession number: MT773374).

The complete cp genome of DHP displayed the typical quadripartite structure of most angiosperm cp genomes, including the large single-copy (LSC, 86,633 bp), the small single-copy (SSC, 18,282 bp) and a pair of inverted repeats (IRa and IRb, 26,081 bp). The GC contents of the LSC, SSC, and IR regions individually, and of the cp genome as a whole, are 35.31, 30.53, 42.95 and 37.29%, respectively. It encoded a total of 113 unique genes, of which 22 were duplicated in the IR regions (ycf15 has four duplicates). Out of the 113 genes, there were 81 protein-coding genes, 28 tRNA genes, and 4 rRNA genes. 18 genes contained introns, 16 (twelve protein-coding and four tRNA genes) of which contained one intron and two of which (ycf3 and clpP) contained two introns.

A phylogenetic analysis was performed based on complete cp genomes from 37 *Camellia* species representing two subgenera with *Apterosperma oblata* as the outgroup species. All of these 38 complete cp genomes were aligned by the MAFFT version 7 software (Katoh and Standley 2013). Bayesian inference analysis was carried out in MrBayes v.3.2.2 (Ronquist et al. 2012) based on the GTR substitution model, which was selected by the Akaike Information Criterion (Posada and Buckley 2004) as implemented in the program Modeltest v.3.7 (Posada and Crandall 1998). The result strongly supported that DHP was closely related to *C. sinensis* cv. *Longjing* (Figure 1). In addition, our results also showed that not all subgenus *Thea* or subgenus *Camellia* were individually clustered according to Ming’s classification (Ming 2000), reflecting highly intricate phylogeny and taxonomy of *Camellia* (Lu et al. 2012).

**Figure 1.**
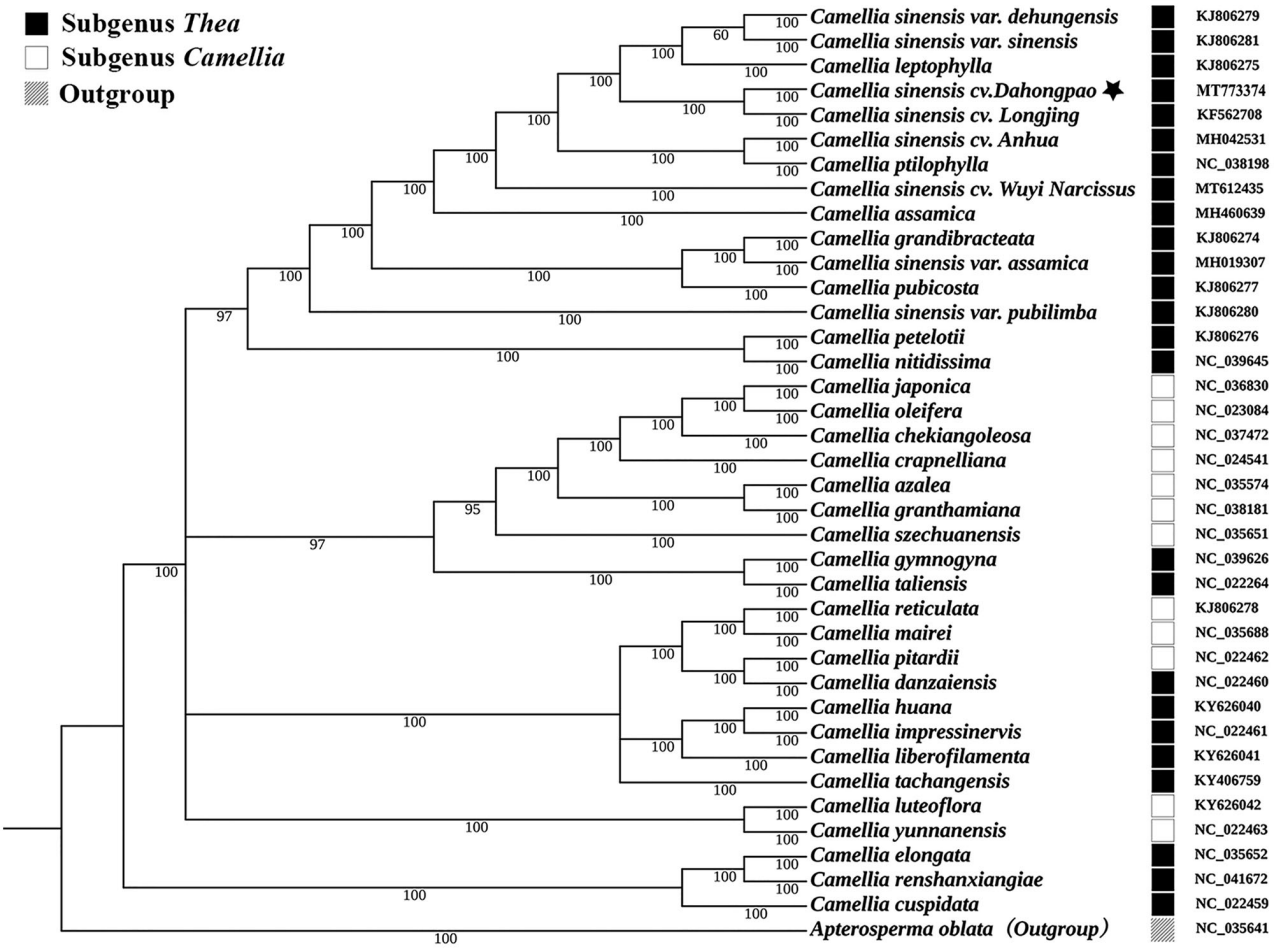
Bayesian inference analysis based on 38 complete chloroplast genomes with *Apterosperma oblata* as an outgroup. The bootstrap support values (>50%) were shown above the branches. The position of *Camellia sinensis* cv. *Dahonpao* was marked with an asterisk and GenBank accession numbers were listed behind each species name. Species of subgenus *Thea* and subgenus *Camellia* were mark with the black box and the white box, respectively.

## Data Availability

The data that support the findings of this study are openly available in the US National Center for Biotechnology Information (NCBI database) at https://www.ncbi.nlm.nih.gov/, reference number: MT773374.
